# A neurosurgical reappraisal of President Lincoln’s fatal cranial injury

**DOI:** 10.1007/s10143-025-03984-2

**Published:** 2025-12-08

**Authors:** Mustafa Ismail, Brian Saway, Mohammed Bani Saad, Zachary Hubbard, Robert J. Weil, Alejandro M. Spiotta

**Affiliations:** 1 Department of Neurosurgery, Medical University of South Carolina, 96 Jonathan Lucas Street, Charleston, SC 29425 USA; 2 Al Kindy Teaching Hospital, Baghdad, Iraq

**Keywords:** Penetrating brain injury, Intracranial pressure, Forensic neurosurgery, Ballistic trajectory analysis, Artificial intelligence

## Abstract

President Abraham Abraham Lincoln’s assassination in April 1865 remains one of the most scrutinized cases in forensic history. We re-examined his fatal head wound through the lens of modern neurosurgery, focusing on projectile trajectory, biomechanics, skull base anatomy, fluid percussion, and periorbital injury, and including the use of artificial intelligence search. A systematic review was conducted using PubMed (*n* = 19) and Scopus (*n* = 12), along with 5 additional sources available on the Internet. Following PRISMA guidelines, 3 original studies were included. Historical autopsy documents were analyzed alongside contemporary literature. Generative AI models (Gemini, Claude, Perplexity, ChatGPT) were used to assist in triangulating ballistic and clinical interpretations. Separate autopsy reports by Woodward, Curtis, and Stone confirm a left occipital entry wound, with the bullet traveling posterior-anterior through the left hemisphere and lodging above the corpus striatum. The absence of midline crossover is consistent across modern reconstructions. Bilateral orbital fractures and right-sided ecchymosis are best attributed to heightened pressure-wave dynamics, not to direct trauma or contrecoup effects. Lincoln’s injury represents a non-survivable low-velocity penetrating brain injury. The forensic and neurosurgical evidence supports a strictly left-sided trajectory, possible basal skull fractures, and pressure-driven orbital injury.

**Supplementary Information:**

The online version contains supplementary material available at 10.1007/s10143-025-03984-2.

## Introduction

As Sherlock Holmes—under the pen of Arthur Conan Doyle—remarked, “There is nothing more deceptive than an obvious fact.”

On April 14, 1865, President Abraham Lincoln was mortally wounded by a low-velocity 0.44 caliber round fired at close range by John Wilkes Booth during a performance at Ford’s Theatre in Washington, D.C. The bullet entered the left occipital region approximately one inch left of midline, traversed the left posterior cerebrum (Occipital lobe), and lodged near the anterior portion of the left corpus striatum (Basal ganglia) [1]​. While modern trauma paradigms emphasize stabilization, imaging, and decompression in managing penetrating craniocerebral injury​, Lincoln’s care was dictated by 19th-century limitations, primarily guided by intuitive observation and crude probing techniques using early instruments such as the Nélaton probe, a metallic probe with a porcelain tip, first developed in 1862 [2–5].

Controversy has persisted regarding both the trajectory of the bullet and the pathophysiological mechanism underlying Lincoln’s striking right orbital ecchymosis. Contemporaneous accounts from primary autopsy physicians, including Drs. Woodward, Curtis, and Stone maintained that the projectile remained in the left hemisphere; later, contradictory testimony suggested a cross-midline injury [1, 3]. More recent forensic analysis based on ballistic mechanics, eyewitness reconstructions, and architectural reexamination of the Presidential box at Ford’s Theatre supports the original autopsy findings [5, 6].

The distinctive right orbital ecchymosis and bilateral orbital plate fractures are reinterpreted through modern biomechanical and neuropathologic principles, suggesting a contrecoup or transient intracranial pressure wave phenomenon instead of direct ballistic trauma. Here, we reexamine Lincoln’s cranial wound through a modern neurosurgical lens, assessing projectile trajectory, injury biomechanics, and periorbital findings using contemporary diagnostic and forensic standards and apply AI analyses to re-assess the likely trajectory of the bullet that killed President Lincoln.

## Methods

A systematic review of the literature was conducted following The Preferred Reporting Items for Systematic Reviews and Meta-Analyses (PRISMA, 2020) [7], using PubMed and Scopus databases. The search strategy included combinations of the following keywords: *“Abraham Lincoln*,*” “assassination*,*” “gunshot wound*,*” “head trauma*,*” “ballistic trajectory*,*” “neuroforensics*,*”* and *“postmortem examination.”* Boolean operators (AND, OR) were applied to refine the search and capture relevant results. The search was not restricted by publication year but was limited to articles published in English.

In addition to database searches, the reference lists of all included articles were manually reviewed to identify further relevant publications not captured in the initial queries. The inclusion criteria were English-language original reports. Peer-reviewed articles or historical reports are judged to be reliable based on authorship, primary source documentation, or institutional provenance.

The exclusion criteria were non-English publications, anecdotal or speculative accounts without historical or scientific substantiation. In addition to popular media, opinion pieces, or dramatizations lacking forensic or clinical rigor.

Following identification and selection, a narrative review was performed to synthesize the data from the included literature. Emphasis was placed on autopsy descriptions, ballistic path reconstruction, orbital injury mechanisms, and modern reinterpretations of historical findings through a neurosurgical lens.

Additionally, advanced generative artificial intelligence (AI) models were utilized to help organize, synthesize, and interpret historical and medical literature. Specifically, Google Gemin 2.5 pro, Claude Sonnet 4, Perplexity free version, and Open AI ChatGPT o3 Pro were used to extract relevant information, cross-reference primary sources, and aid in the critical appraisal of forensic and neurosurgical findings. These AI tools enhanced the integration of complex multidisciplinary data, improving the accuracy and coherence of the narrative review.

## Results

### Study selection

**Fig. 1:**
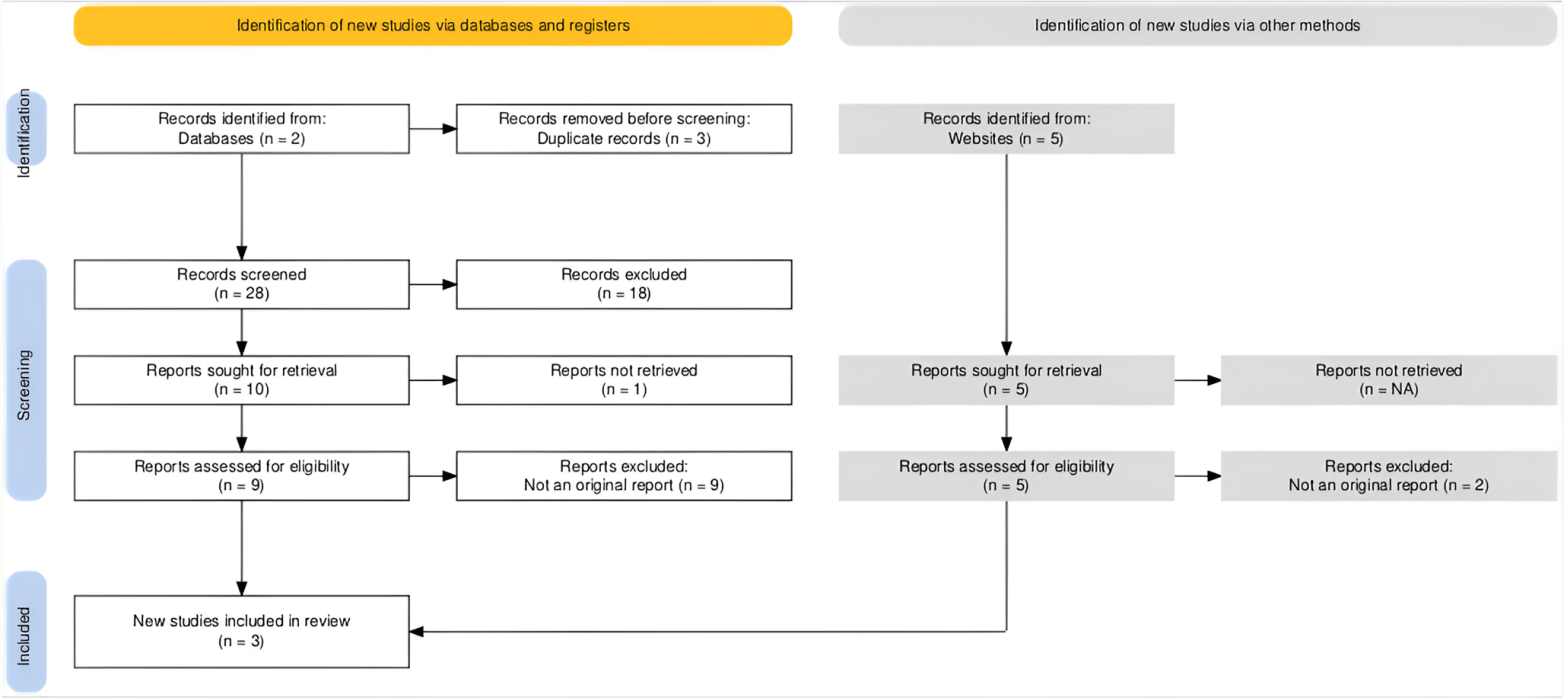
PRISMA flow diagram of the included articles

Following PRISMA 2020 guidelines, a total of 31 records were identified—19 from PubMed, 12 from Scopus, and 5 from additional Internet-only sources. After duplicate removal and screening, 10 reports were reviewed in full and assessed for eligibility. Ultimately, three original and relevant studies were included in the final analysis, as visualized in the PRISMA flow diagram (Fig. 1).

### Wound trajectory and skull findings

Based on the primary historical sources [1–3, 5–8] and reconstructed modern forensic analyses [6]​, the bullet entered approximately one inch to the left of the midline of President Lincoln’s occipital bone. The internal table of the skull showed a beveled, larger opening compared to the external table, consistent with a typical, inward low-velocity gunshot wound. The bullet traveled anteriorly, traversing the left occipital lobe and left lateral ventricle and lodging just above the anterior portion of the left corpus striatum (Table 1).

**Table 1:** Direct available reports from the literature with their analysis

#	Examiner & role†	Date & time of examination/setting	When account was written/published	Wound description (entry site)	Ballistic path reconstructed	Neuro-forensic observations	Notable orbital findings	Neurosurgical/clinical perspective
1	**Dr Charles A. Leale** – 23-y-old Assistant Surgeon, U.S. Army; first physician on scene at Ford’s Theatre [5]	~ 22:15 h, 14 Apr 1865 (inside presidential box → helped move Lincoln to Petersen House)	Bed-side notes 15 Apr 1865; formal report to Surgeon-General 1867, rediscovered 2012	1 in below superior curved line of occiput, 1.5 in left of mid-line	Smooth track forward through occiput → deep cerebrum; no exit	Immediate coma, agonal breathing; digital & Nélaton-probe exploration; recognized mortal prognosis	Progressive right-eye ecchymosis & proptosis developing hours after injury	Early recognition of rising ICP; brief relief after clot drainage but declared wound inevitably fatal
2	**Dr Robert K. Stone** – Lincoln’s long-time personal physician (civilian MD, Washington DC) [3]	Arrived ~ 23:00 h, 14 Apr; remained bedside until death 07:22 h, 15 Apr 1865 (Petersen House)	Autopsy notes & bedside diary 15 Apr 1865; excerpts printed in medical journals 1865–1870	Circular entry ~ 1 in left of mid-line, 3 in from external auditory meatus; internal table larger	Forward/upward through left thalamus & corpus striatum; lodged anterior to L corpus striatum	Brain “pultaceous”; massive ventricular hemorrhage; bullet & bone fragments along track	Bilateral orbital ecchymosis & protrusion attributed to intracranial pressure, not external trauma	Emphasized pressure-wave transmission causing orbital-plate fractures; confirmed rimmed. lethality
3	**Dr Edward G. Curtis** – Assistant Surgeon, U.S. Army; one of two primary autopsy surgeons [1]	Autopsy began 12:10 h, 15 Apr 1865, Guest Room, White House	Detailed letter to his mother 22 Apr 1865; first published 19th cent.	Entry slightly left of mid-line at occiput; track “full of clots”	Occiput → center of brain → left ventricle; bullet found when whole brain removed	Ventricles filled with clot; brain weight recorded; the described sound of bullet falling into basin	“Eyes somewhat protuberant”; orbits filled with blood; both orbital plates fractured upward	Classic description of closed-cranium low-velocity track; pressure-induced orbital blow-out noted
4	**Dr Joseph J. Woodward** – Assistant Surgeon & pathologist, Army Medical Museum; co-autopsy surgeon [1]	Same autopsy session (15 Apr 1865)	Official autopsy report to Surgeon-General, dated 15 Apr 1865; widely reproduced thereafter	Entry 1 in left of mid-line, just above left lateral sinus; scalp thickened by hemorrhage	Through left posterior cerebrum → left lateral ventricle → lodged above anterior L corpus striatum	Track “pultaceous & livid”; thick sub-dural clots, ventricles gorged; orbital plates fractured	Both orbital plates comminuted & pushed upward; orbits “gorged with blood”; dura intact.	Interpreted bilateral orbital fractures as pressure-wave venting, not exit; highlighted low-velocity closed-skull pattern.

Critically, across all forensic reviews, the projectile is consistently documented to have remained within the left cerebral hemisphere without crossing the midline​​, despite early but contradictory suggestions by Dr. Taft [8]. Modern ballistic principles, combined with the absence of an anterior exit wound and the symmetry of parenchymal destruction described in autopsy reports, reinforce the trajectory as straight, left-sided.

### Bullet recovery and implication of skull base fractures

Notably, the bullet fell freely from Lincoln’s extracted brain during the autopsy (through Curtis’s fingers into a basin​) [1, 8]. From a neurosurgical perspective, this observation may suggest an associated skull base fracture.

An important distinction must be made when interpreting the bullet’s final position. If, after the cerebrum was lifted out of the cranial vault, the projectile rolled posteriorly along the curvature of the skull base, this behavior indicates it had been resting beneath the brain on the anterior cranial floor. A bullet truly embedded in parenchyma would instead have dropped straight down once the supporting tissue was removed, without rolling. The autopsy description of the projectile “rolling” into the basin during the autopsy, with the subject supine on the dissecting table, therefore, supports a conclusion that it lay on the anterior skull base, not within cerebral tissue. This can be explained by the melting energy of the bullet. Initially, it lodged with the parenchyma; however, as hours passed, it changed its position downward to the skull base.

**Fig. 2:**
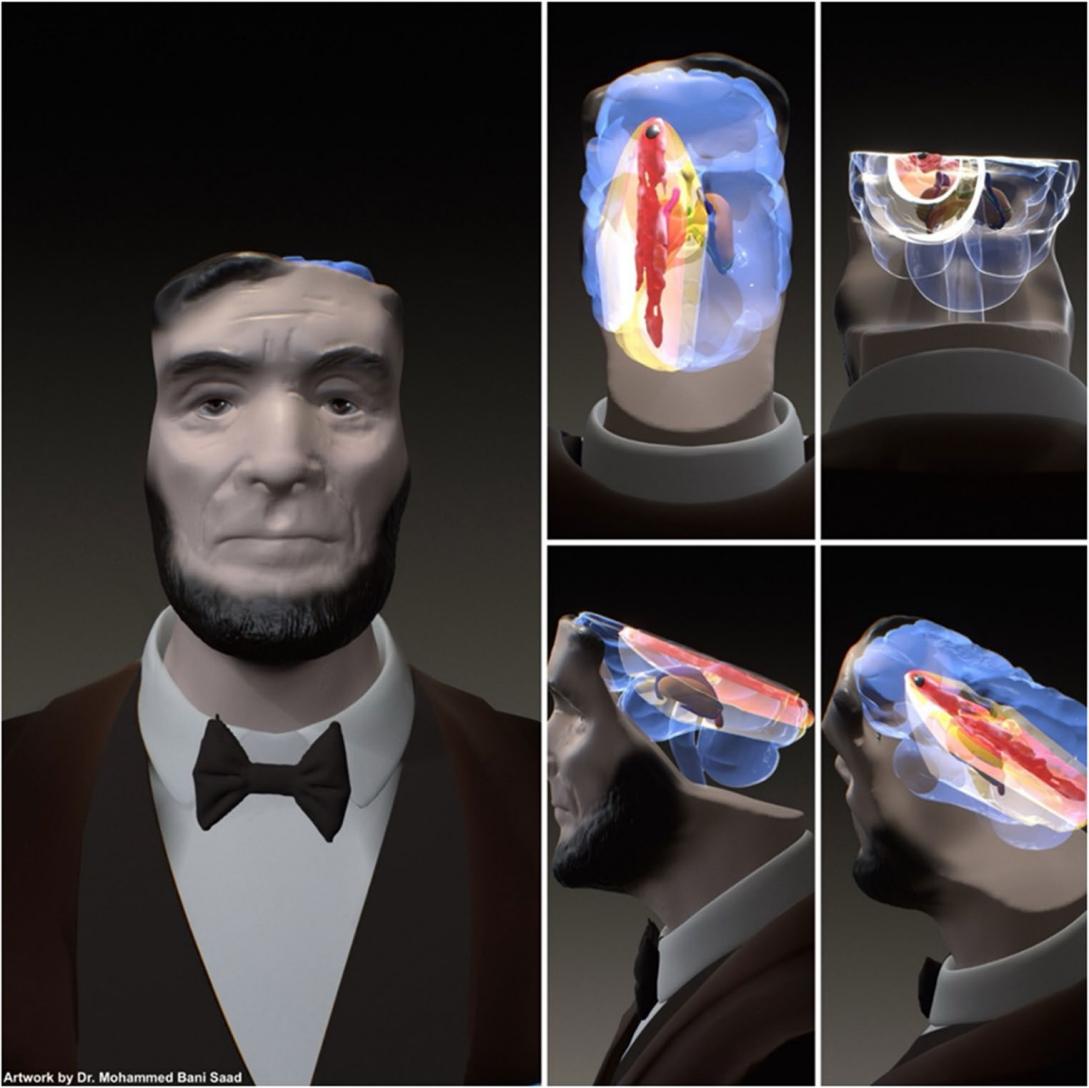
Schematic representation of the bullet’s trajectory with the surrounding blast.

**Fig. 3:**
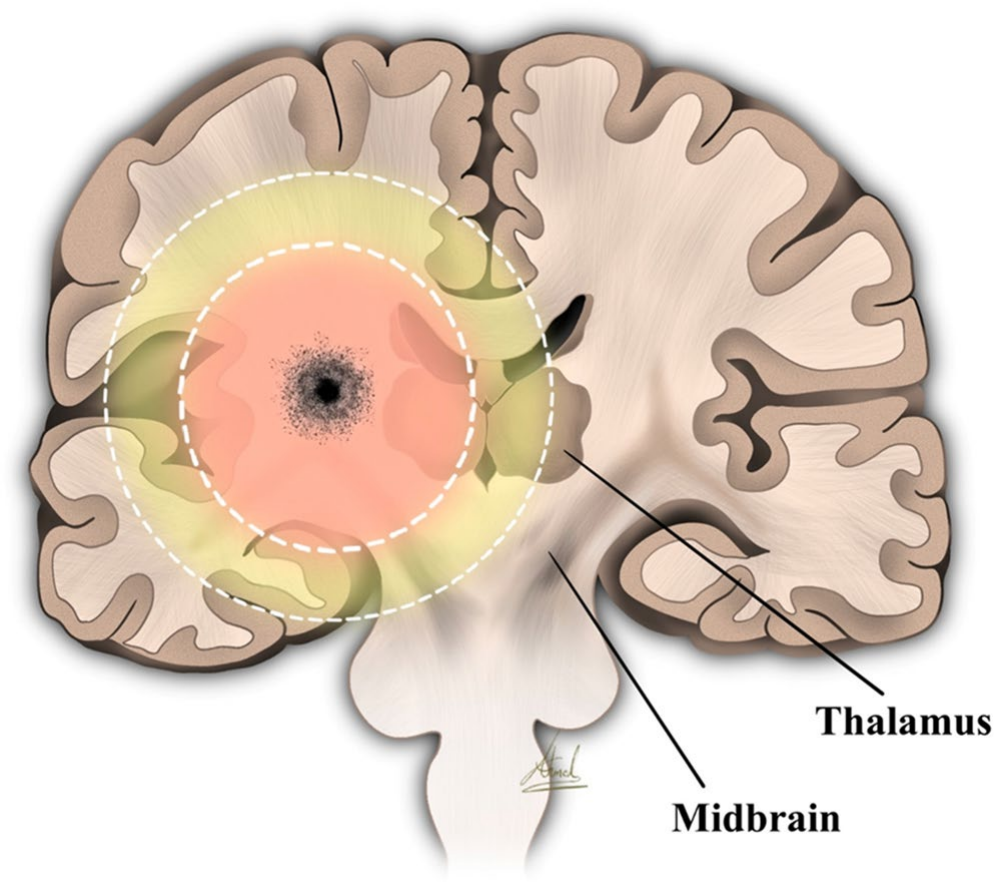
An illustration depicting a coronal view of the bullet trajectory transversing corpus striatum showing inner circle 3 cm and outer circle 4 cm, the effect of bullet blast

Moreover, the immediate loss of consciousness of the president after the shot may indicate the bullet may have transected the midbrain region. This hint can add to the evidence about the location which is near/on the skull base. Based on low-velocity civilian handgun ballistics, the transient cavity can enlarge to about 3–4 cm in radius—stretching and pressurizing surrounding brain tissue—and its pressure spike may propagate far enough to injure deep structures such as the thalamus and rostral midbrain, producing diffuse damage that exceeds the visible missile path (Figs. 2 and 3) [9] .

### Interrogating the evidence with AI

Generative AI models (Gemini, Claude, Perplexity, and ChatGPT) were used to independently analyze the forensic data. While interpretations varied slightly, all models supported a fatal low-velocity gunshot to the left occiput, with ChatGPT providing the most anatomically consistent synthesis aligning with historical and current neurosurgical interpretations of the contemporaneous findings (Table 2).

**Table 2:** A comparison between different AI models analyzing lincoln’s assassination from a neurosurgical perspective

AI model	Bullet trajectory	Skull-base fracture implicated?	Explanation for right-sided “raccoon eye”	Ultimate neurosurgical conclusion
**Gemini**	Crosses midline: enters left occiput, lodges behind right orbit.	Yes – anterior-cranial-fossa/orbital-roof fracture.	Venous blood tracks through basilar fracture into periorbital tissue.	Fatal penetrating injury; death from massive intracranial pressure and brain-stem herniation.
**Claude**	Remains left sided: occiput → left lateral ventricle → above left corpus striatum.	Mentioned venous-sinus laceration; fracture not emphasized.	Not detailed.	Catastrophic left-hemisphere destruction with intraventricular hemorrhage; inevitable herniation.
**Perplexity**	Remains left sided; same route as Claude.	Yes – transverse-sinus/orbital-plate fractures.	Pressure-wave fractures orbital roofs, causing bilateral ecchymosis (right > left).	Fatal uncal herniation and brain-stem compression; modern care would not alter outcome.
**ChatGPT**	Best evidence supports a strictly left-hemisphere path: bullet enters left occiput, travels anterior-superior through left occipital lobe and lateral ventricle, and stops just in front of the left basal ganglia; no midline crossover.	Highly likely: a posterior skull-base fracture (transverse-sinus region) explains the bullet’s post-extraction fall and contributes to venous bleeding.	Bilateral—but right-dominant—periorbital ecchymosis results from pressure-wave–induced orbital-roof fractures, not direct bullet passage.	Injury resulted in irreversible brain destruction, massive hemorrhage, rapidly escalating intracranial pressure, and brain-stem herniation; survival would remain virtually impossible even with today’s neurosurgical capabilities.

### Orbital fractures and right-sided “raccoon eye”

Both Woodward and Stone detailed the presence of bilateral orbital plate fractures in President Lincoln, with fragments displaced upward toward the brain; in addition, both orbits were described as massively engorged with blood [1, 3, 8]. Clinically, this manifested as right-sided periorbital ecchymosis—the so-called “raccoon eye”—which was observed by both Leale and Stone in Lincoln’s final hours. Importantly, this finding cannot be attributed to external trauma or direct bullet passage, as the entry wound was posterior and there was no evidence of anterior cranial penetration. Instead, the pattern is consistent with a possibility of basal skull fracture, in which venous blood tracks along fascial planes into the periorbital tissues—a well-recognized neurosurgical indicator of skull base injury. Supporting this interpretation, modern biomechanical studies have demonstrated that low-velocity gunshot wounds can generate a transient intracranial pressure wave, which preferentially disrupts fragile structures like the orbital roof [8]. Experimental data from primate and human cadaveric models confirm that sudden expansion of intracranial volume, followed by rapid collapse, can produce a vacuum effect capable of drawing the orbital plates inward, leading to fractures without direct impact. Therefore, the bilateral orbital fractures, with more pronounced clinical signs on the right, are best understood resulting from elevated intracranial pressure translated as a potent power wave, rather than the result of contrecoup injury or anterior impact. From a modern neurosurgical perspective, the evidence supports that the bullet’s path remained exclusively within the left hemisphere, as originally documented by Woodward, Curtis, and Stone. The bullet’s dislodgment and rolling over the skull base during brain removal most likely reflects that the bullet has fractured the skull base bones which may result in the raccoon eye. Thus, the most likely explanation for the right orbital ecchymosis and bilateral orbital fractures is pressure-wave dynamics and vacuum collapse, not direct ballistic trajectory. The ultimate cause of death was intracranial hypertension, driven by intraventricular and subdural hemorrhage, culminating in brainstem herniation—a mechanism now universally recognized in both historical autopsy reports and modern trauma paradigms [1–3, 5, 8].

### Timeline for right eye ecchymosis

President Lincoln was shot at approximately 10:15 p.m. on Friday, April 14, 1865. At 10:30 p.m., an initial assessment at the Petersen House showed a faint bruise on Lincoln’s left upper eyelid, with the rest of the orbits appearing normal. By around 11:00 p.m., increasing cerebral venous pressure led to swift changes in the right orbit: proptosis of the globe, a surrounding crimson discoloration, and an expansion of bruising above the supraorbital ridge and below the infraorbital foramen, creating a classic raccoon-eye appearance. The left orbit mainly remained unaffected. By midnight, growing swelling and bruising in the right orbit prompted doctors to raise Lincoln’s head and drain occipital clots to alleviate the rising intracranial pressure. At 1:00 a.m., tense eyelids on the right, conjunctival swelling, and worsening discoloration signified ongoing venous congestion due to an underlying basal skull fracture. By dawn on April 15, the right periorbital tissues appeared violet black, distinctly marking the venous blood tracing along fascial planes from a fractured orbital roof under ongoing intracranial hypertension (Fig. 2, Video 1) [5] .

## Discussion

We have re-examined President Abraham Lincoln’s deadly head injury using modern neurosurgical and forensic principles, complimented and extending using AI analysis. By meticulously examining original autopsy records and comparing them with current insights into projectile biomechanics, intracranial pressure, and orbital fracture processes, we propose a unified explanation of the President’s fatal injury.

### Projectile trajectory: revisiting the forensic truth

The bullet’s trajectory is crucial to interpreting Lincoln’s evolving physical findings, the results of wound probing, and death. Accounts from Woodward and Curtis at the April 15th autopsy noted a left occipital entry, about one inch left of midline, with the projectile moving through the left occipital lobe, left lateral ventricle, and lodging anterior to the left corpus striatum [1, 3]. Modern reconstructions of the shooting geometry in Ford’s Theatre support these findings, showing that Booth’s position makes a right-sided entry unlikely [6].

Although Taft and Barnes later proposed that the bullet crossed the midline and ended near the right orbit, this assertion lacks anatomical and ballistic coherence. Their proposal appears to have been influenced by the orbital ecchymosis on the right and the bullet’s postmortem mobility, rather than direct visualization of the projectile’s resting site [10, 11].

Modern neurosurgical consensus confirms that in the absence of an anterior skull fracture on the right or an exit wound, cross-midline migration of a low-velocity projectile is highly unlikely, particularly in the absence of sustained kinetic energy or yaw. In contrast, the ball’s rolling over the skull base bones during brain extraction from the cranial vault at autopsy, as noted by Dr. Curtis, is strongly indicative of an underlying skull base fracture, which may result in raccoon eye, and this also may indicate the bullet did not cross the midline. It was a low-velocity bullet with modest kinetic energy, which can result in a shorter trajectory than the one that is supposed to cross the midline [1, 11].

### Biomechanics of injury: intracranial pressure and tissue destruction

From a biomechanical perspective, Lincoln’s injury presents as a classical example of a low-velocity, closed-cranium gunshot wound causing more localized tissue destruction but compounded by pressure wave propagation and localized pulping of neural tissue. The autopsy descriptions of “pultaceous” parenchyma and ventricular hemorrhage are consistent with pressure-driven disruption along the bullet’s path [1, 12]. As Yan et al. suggested, the primary mechanism of fatality may have been due less to substantive, primary direct destruction of brainstem structures, rather than to secondary, progressive mass effect (blood and swollen brain tissue), with rapid elevation in intracranial pressure (ICP), with herniation and circulatory collapse [11].

The historical practice of repeatedly evacuating clots from the wound, as performed by Dr. Leale and later Stone, serves as an early analog to modern decompressive procedures. Their observation that respiratory function transiently improved following clot removal demonstrates the physiological effect of ICP decompression through the bullet’s tract [5, 13].

If encountered today, management would include airway protection, rapid CT and CTA imaging, broad-spectrum antibiotics, ICP monitoring, and consideration of external ventricular drainage and decompressive craniectomy.

Given the transventricular, deep-basal trajectory with significant intraventricular and subdural hemorrhage, survival—even with advanced neuro-critical care—would remain unlikely. Decompressive surgery might temporarily decrease ICP but would not restore lost brainstem function. This aligns with contemporary outcomes for similar low-velocity penetrating cranial gunshots, in which mortality approaches >90% despite maximal intervention [11, 12, 14–16]. Furthermore, for the early period of pupil reactivity, a rapid-sequence CT followed by immediate posterior decompression or EVD might have delayed herniation, but irreversible bi-hemispheric damage and transventricular tract necrosis would still have rendered recovery impossible [11, 12, 14–16].

### Periorbital findings: from misinterpretation to mechanical insight

The right-sided periorbital ecchymosis—a raccoon eye—and bilateral orbital plate fractures are perhaps the most misinterpreted aspects of Lincoln’s cranial injury. Emphasized by Dr. Stone, these findings were long thought to suggest bullet migration or a contrecoup effect [1, 2]. However, modern biomechanical models suggest a more likely mechanism: a pressure wave venting through the thin orbital roofs.

Lattimer had attempted to simulate skull deformation to explain this, but his methods—using unspecified skulls and vague impact criteria—lacked scientific reproducibility [13]. In contrast, contemporary ballistic studies, as outlined in Nguyen in 2020, demonstrate that low-velocity intracranial projectiles produce a temporary pressure surge, which expands radially and collapses ventrally, with sufficient force to cause orbital plate implosion without external impact [12].

This concept is now supported by both primate and cadaveric model studies, which show that thin cranial structures such as the orbital roofs fail at pressure thresholds well below those required for vault fracture. Thus, the orbital ecchymosis and inwardly displaced bone fragments should not be viewed as evidence of midline bullet crossover but rather as pressure-outlet injury zones—akin to a “release valve” for the massive energy within the closed cranial cavity [6, 12, 17, 18].

In addition to pressure-wave and basal-skull–fracture mechanisms, two additional causes merit mention.

First, Terson’s syndrome—vitreoretinal or intraocular hemorrhage secondary to abrupt ICP elevation could theoretically explain peri-orbital discoloration within minutes of injury. Second, instantaneous right-sided hemiplegia could have precipitated an asymmetric fall in the presidential box, producing minor blunt trauma to the dependent right orbit. While no external abrasions were documented, the hypothesis cannot be entirely excluded. Nevertheless, the absence of external contusion or eyelid laceration, coupled with bilateral orbital-roof fractures at autopsy, continues to favor a skull-base pressure-wave mechanism as the dominant cause.

### Analysis using modern AI tools

Recent advances in generative artificial intelligence AI tools offer a novel means of triangulating complex historical forensic interpretations. When applied to Lincoln’s cranial wound, AI models (Gemini, Claude, Perplexity, and ChatGPT) demonstrated varying levels of concordance with historical findings (see Table 2). Gemini proposed a cross-midline trajectory with lodging behind the right orbit and implicated an anterior fossa fracture to explain the right-sided “raccoon eye”; however, this interpretation contrasts with physical autopsy evidence and forensic reconstructions [3, 6]. Claude and Perplexity both preserved a strictly left-sided bullet path, with Perplexity emphasizing posterior skull-base fractures and pressure-wave–induced orbital fractures leading to bilateral, right-dominant ecchymosis [11, 12]. The most clinically-balanced synthesis, by ChatGPT, aligned most closely with autopsy data and biomechanical modeling, outlining a left-hemisphere-only path, posterior skull-base fracture, pressure-wave-induced orbital injury, and fatal, uncal herniation as the immediate cause of death [1, 14]. Collectively, these AI-driven analyses reinforce that Lincoln’s wound, viewed through modern neurosurgical and forensic standards, represents an unsurvivable, low-velocity, penetrating brain injury with a lethal secondary intracranial hypertensive crisis.

The current study corresponds with an expanding sector in which artificial intelligence is utilized to recover lost or deteriorated historical documents—spanning from AI-restored Greek inscriptions utilizing Ithaca to the virtual unwrapping of carbonized Roman scrolls through Aeneas. These advancements demonstrate the growing intersection of data science, forensics, and digital humanities with medical-historical research [19, 20].

### Historical tools, modern parallels

The Nélaton probe was used on the President to track the bullet’s path. While tactile and subjective, this porcelain-tipped tool was the only option at the time used for objective intraoperative bullet confirmation, effectively used by Drs. Leale, Taft, and Barnes [4, 5]. Lacking imaging, it functioned like modern surgical navigation but without sterility or precision. Their practice of probing the wound without antiseptic technique—though unacceptable today—was consistent with standard of care in 1865. Joseph Lister’s antiseptic theory had only just been published, and germ theory was still controversial [14].

Overall, contemporary forensic analyses, combined with a more modern understanding of bullet trajectories and intracranial pressure dynamics, complimented by AI analysis, suggest strongly that Lincoln’s bullet remained entirely within the left hemisphere, with no evidence of midline crossover. The bullet’s fall at autopsy indicates a posterior skull base fracture, not anterior cranial involvement. The orbital fractures and right-sided ecchymosis resulted from pressure-wave venting, not direct bullet migration or contrecoup trauma.

The limitations of the current study include its reliance on historical documents, artist sketches, and secondary descriptions rather than preserved physical specimens. Although the integration of AI tools is innovative, it is limited by the accuracy of the underlying digital sources and cannot replace direct forensic re-examination. Therefore, the proposed biomechanical interpretations should be regarded as probabilistic reconstructions rather than definitive forensic conclusions.

## Conclusion

President Lincoln’s wound likely indicates a fatal low-velocity brain injury from a mostly left-sided bullet trajectory, possibly involving skull-base and orbital pressure changes. Modern care could have temporarily reduced intracranial hypertension, but significant recovery remained unlikely.

## Supplementary Information

Below is the link to the electronic supplementary material.

ESM 1 (MP4 576 KB)

## Data Availability

No datasets were generated or analysed during the current study.
